# Experimental factors affecting the robustness of DNA methylation analysis

**DOI:** 10.1038/srep33936

**Published:** 2016-09-27

**Authors:** Heidi D. Pharo, Hilde Honne, Hege M. Vedeld, Christina Dahl, Kim Andresen, Knut Liestøl, Marine Jeanmougin, Per Guldberg, Guro E. Lind

**Affiliations:** 1 Department of Molecular Oncology, Institute for Cancer Research, Oslo University Hospital, the Norwegian Radium Hospital, Oslo, Norway; 2 KG Jebsen Colorectal Cancer Research Centre, Oslo University Hospital, Oslo, Norway; 3 Centre for Cancer Biomedicine, Faculty of Medicine, University of Oslo, Oslo, Norway; 4 Department of Biosciences, The Faculty of Mathematics and Natural Sciences, University of Oslo, Oslo, Norway; 5 Danish Cancer Society Research Center, Copenhagen, Denmark; 6 Department of Informatics, University of Oslo, Oslo, Norway

## Abstract

Diverging methylation frequencies are often reported for the same locus in the same disease, underscoring the need for limiting technical variability in DNA methylation analyses. We have investigated seven likely sources of variability at different steps of bisulfite PCR-based DNA methylation analyses using a fully automated quantitative methylation-specific PCR setup of six gene promoters across 20 colon cancer cell lines. Based on >15,000 individual PCRs, all tested parameters affected the normalized percent of methylated reference (PMR) differences, with a fourfold varying magnitude. Additionally, large variations were observed across the six genes analyzed. The highest variation was seen using single-copy genes as reference for normalization, followed by different amounts of template in the PCR, different amounts of DNA in the bisulfite reaction, and storage of bisulfite converted samples. Finally, when a highly standardized pipeline was repeated, the difference in PMR value for the same assay in the same cell line was on average limited to five (on a 0–100 scale). In conclusion, a standardized pipeline is essential for consistent methylation results, where parameters are kept constant for all samples. Nevertheless, a certain level of variation in methylation values must be expected, underscoring the need for careful interpretation of data.

Aberrant DNA methylation is associated with various diseases^1^, and growing interest in this mechanism, as well as technological advances, have resulted in the identification of a vast number of disease-specific methylated loci^2^. However, diverging methylation frequencies are commonly reported for the same locus in the same type of disease, also among studies with similar disease subtype composition. This is exemplified by a systematic comparison of *MGMT* promoter methylation frequencies in gliomas, reported to range from 19% to 97% across almost 100 publications^3^. Furthermore, diverging methylation frequencies have been reported for various genes in several types of cancer from studies using the same method for DNA methylation analysis^4^,^5^,^6^, underscoring the need for standardization.

Numerous methods for DNA methylation analysis exist, and the majority of these consist of a DNA pre-treatment step, and an analytical step to determine methylation status. A frequently used combination involves sodium bisulfite conversion of DNA, followed by PCR-based methylation analysis of the loci of interest. Examples of conventional and commonly used methylation analysis variants include methylation-specific PCR (MSP)^7^, quantitative methylation-specific PCR (qMSP; also called MethyLight)^8^, bisulfite sequencing^9^, and pyrosequencing^10^. More recently, also the digital PCR (dPCR) technology has been applied for methylation analyses^11^. The abovementioned variants can be used to determine the methylation status at a limited number of preselected loci. On the other hand, large-scale methylation analyses such as reduced representation bisulfite sequencing (RRBS)^12^, whole genome bisulfite sequencing (WGBS), and various methylation arrays (Illumina), enable detection of the methylation pattern at the genome level. The choice of method will depend on the aims of a given study.

Here we have analyzed the extent of variability that can arise from bisulfite PCR-based DNA methylation analyses, using qMSP as a representative method. Our aim was to identify important contributors of technical variability in the various steps of the pipeline, and to provide guidelines for more robust analyses.

## Materials and Methods

### Cell line DNA

DNA from the following 20 colon cancer cell lines was used to investigate promoter methylation status; Co115, Colo205, Colo320, EB, FRI, HCT15, HCT116, HT29, IS1, IS3, KM12, LS1034, LS174T, NCI-H508, RKO, SW480, SW620, SW948, TC71, and V9P. HCT15, HCT116, NCI-H508, RKO, SW620, and SW948 have been purchased from the American Type Culture Collection (ATCC), and Colo205 and KM12 from the Charles River Laboratories. The rest of the cell lines have kindly been provided by Professor Richard Hamelin (INSERM, Paris, France). DNA was isolated using either a standard phenol/chloroform protocol, or a magnetic beads approach (Maxwell^®^ 16 System, Promega). For each cell line, the same DNA stock was used for all analyses. All cell lines have been tested by short tandem repeat (STR) profiling using the AmpFLSTR Identifiler PCR Amplification Kit (Life Technologies). Commercial lines have been authenticated based on comparisons with available STR profiles from the American Type Culture Collection (ATCC) and the German Collection of Microorganisms and Cell cultures^13^. All cell lines tested negative for mycoplasma infection (MycoAlert Mycoplasma Detection Assay and Lucetta Luminometer, Lonza).

### DNA methylation analysis

DNA from all cell lines was subjected to bisulfite conversion with either the EpiTect Bisulfite Kit (Qiagen), or the EZ DNA Methylation-Gold Kit (Zymo Research) according to the manufacturers’ protocols. The MJ Mini Personal Thermal Cycler (Bio-Rad Laboratories) was used to perform the conversion reactions. For samples converted with the Qiagen kit, purification and elution was done automatically in the QIAcube System (Qiagen). DNA concentrations were measured both before and after bisulfite conversion using a NanoDrop 1000 Spectrophotometer (Thermo Fisher Scientific).

The promoter methylation status of the six genes *CNRIP1, MGMT, SEPT9, SFRP1, SPG20*, and *VIM* was analyzed by qMSP in accordance with the MIQE-guidelines. Primers were purchased from BioNordika Bergman, and probes from Life Technologies (see Supplementary Table S1 for sequence information). The EpMotion 5075 pipetting robot (Eppendorf) was used for pipetting samples and mastermix. All samples were run in triplicates in 384-well plates. The total reaction volume per well was 20 μl, including 0.9 μM of each primer, 0.2 μM probe, 1xTaqMan^®^ Universal Master Mix II – no UNG (Thermo Fisher), and bisulfite treated DNA template. The six assays were analyzed in singleplex reactions. In addition to the cell line DNA, each plate contained two methylation-positive controls (commercially available CpGenome Universal Methylated DNA; IVD; Merck Millipore), a non-template control (water), a methylation-negative control (bisulfite treated DNA from healthy donors), and a standard curve consisting of five-fold dilutions of IVD (32.5–0.052 ng). The median quantity (of triplicates) of each of the two methylation-positive controls were averaged and used in calculations of the percent of methylated reference (PMR) values (see below). The qMSPs were run on a 7900HT Fast Real-Time PCR System (Life Technologies), and the amplification involved an initial 10 min at 95 °C, followed by 45 cycles of 15 sec at 95 °C and 1 min at 60 °C. The 7900HT Sequence Detection System version 2.3 (SDS2.3; Life Technologies) was used to analyze the results, and the median of quantities was used for calculations. Samples with C_q_-value ≥ 35.0 were censored according to the manufacturer’s recommendations. The percentage of methylated molecules per sample (PMR value) was calculated by dividing the normalized quantity (investigated assay/reference) of the samples by the normalized quantity of the methylation positive controls, and multiplying by 100 14. ALU was used as a default reference for normalization.

### Experimental design

**Figure 1:**
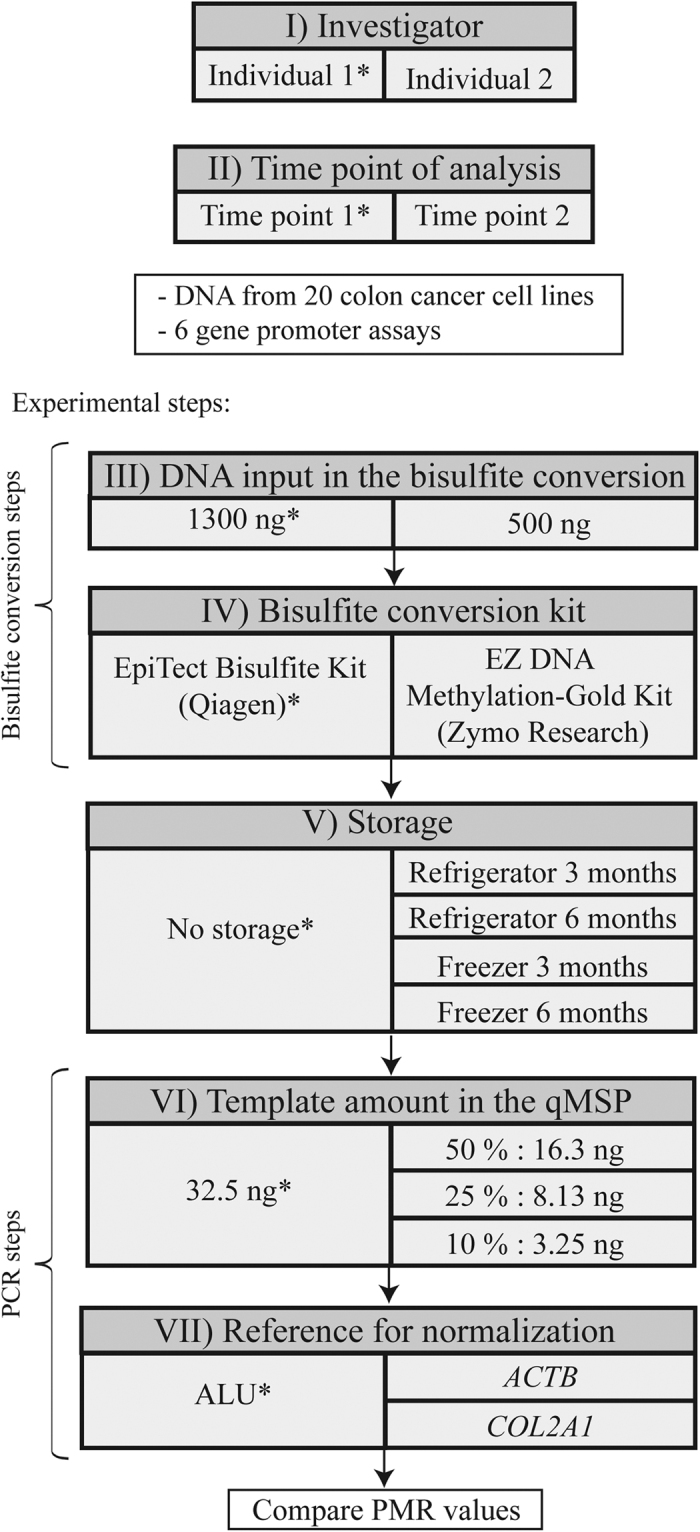
Experimental overview of the investigated sources of variability. Roman numerals represent the investigated sources of variability. Left side: default parameters, marked by an asterisk (*). Right side: alternative parameters. Alternative parameters were tested individually with two exceptions; first, both the default and the alternative input amount in the bisulfite conversion (III) were tested with both bisulfite kits (IV), and second, for testing of storage (V), DNA was bisulfite converted in parallel with the two kits. For Zymo calculations, 500 ng input was used as default parameter. Abbreviations: PMR, percent of methylated reference; qMSP, quantitative methylation-specific PCR.

In the present study, qMSP is the representative method for investigating potential sources of variability in bisulfite PCR-based methylation analyses. Figure 1 illustrates the individual sources of variability (marked with roman numerals) that have been tested, including; I) investigator, II) time point of analysis, III) DNA input amount in the bisulfite conversion, IV) bisulfite conversion kit, V) storage (of bisulfite converted DNA), VI) template amount in the qMSP, and VII) reference for normalization. For each source of variability, one or more alternative parameter(s) were tested against a default (marked with an asterisk in Fig. 1). Alternative parameters were tested individually: when an alternative parameter was chosen for one given source of variability, default parameters were used for the rest of the pipeline (exceptions are indicated in the legend of Fig. 1).

### I–II) Investigator and time point of analysis

The pipeline in Fig. 1 was performed by two different investigators in the same lab (I), and one of the investigators performed the pipeline twice (II).

### III–IV) Bisulfite conversion steps – DNA input and type of kit

For the bisulfite conversion, two bisulfite kits were tested (IV); the EpiTect Bisulfite Kit (Qiagen; default) and the EZ DNA Methylation-Gold Kit (Zymo), each with two different amounts of input DNA (III); 1300 ng and 500 ng (within the recommended range for the Qiagen kit and the Zymo kit, respectively). For the latter, two parallel rounds of bisulfite conversion were performed to have enough template for qMSP analyses. The elution volume was 40 μl for all reactions. The bisulfite converted DNA was diluted to 10.83 ng/μl before qMSP analysis, in order to limit inaccuracies related to pipetting of small volumes of concentrated DNA.

### V) Storage

Bisulfite converted DNA was analyzed either immediately by qMSP (default), or stored in a freezer or refrigerator for three or six months before analysis (V). In the freezer, DNA converted with the Qiagen kit was stored at −20 °C, and DNA converted with the Zymo kit at −80 °C, according to the specifications in the manufacturers’ protocol. All storage-related experiments were performed twice.

### VI–VII) PCR steps – template amount and reference for normalization

For the qMSPs, a theoretical template amount of 32.5 ng bisulfite converted DNA was used (default; VI). Additionally, three alternative template amounts were tested; 16.3 ng (50% of default), 8.13 ng (25% of default) and 3.25 ng (10% of default). ALU was used as the default reference for normalization, with *ACTB* and *COL2A1* as alternatives (VII).

### Statistics and calculation of variability

The difference in PMR values between experiments with default and alternative parameters, ΔPMR = PMR_default_ − PMR_alternative_, was calculated per gene and per cell line. High PMR values will contribute with higher ΔPMR than low PMR values (see Bland-Altman-plots and “Mean versus SD”-plots; Supplementary Figs S1 and S2). To correct for this, the ΔPMRs were divided by the PMR_default_ (ΔPMR/PMR_default_) resulting in a normalized PMR difference. The results have been processed in Excel (Microsoft Excel 2007) and in R 3.2.2, and visualized by GraphPad Prism 6 (GraphPad Software) and by Adobe Illustrator CS4 (Adobe Systems).

To investigate whether the normalized PMR differences varied between the six individual promoter regions analyzed, two statistical analyses were conducted. First, simulations were used to determine whether genes were consequently ranked among the highest or lowest (based on the sum of the absolute normalized PMR differences per gene). For each round of analysis, ranks from 1 to 6 were randomly simulated for the six genes, where 1 was given to the gene with the lowest normalized PMR difference, and 6 to the gene with the highest. Using this procedure, 10,000 datasets were simulated (R 3.2.2), generating a distribution of the rank sums, under the null hypothesis of randomly distributed ranks. The actual observed sum of ranks for the genes was then compared to this distribution, and the probability of observing a higher or lower rank sum was estimated. Second, to investigate potential variation in the normalized PMR differences per gene assay for the individual experimental parameters (*e.g. ‘*6 months storage in freezer’), the coefficient of variation (CV; *i.e*. the standard deviation of the normalized PMR differences over the mean), was calculated across assays for all parameters (R 3.2.2).

Finally, to estimate the expected level of variation for individual PMR values from repeated analyses, PMR values from the comparison ‘different time points of analyses’ (source of variability II; Fig. 1) were used. The absolute differences between two corresponding PMR values (*i.e.* for the same gene in the same cell line) were calculated (R 3.2.2).

## Results

### Main contributors of technical variability in bisulfite PCR-based DNA methylation analyses

**Figure 2:**
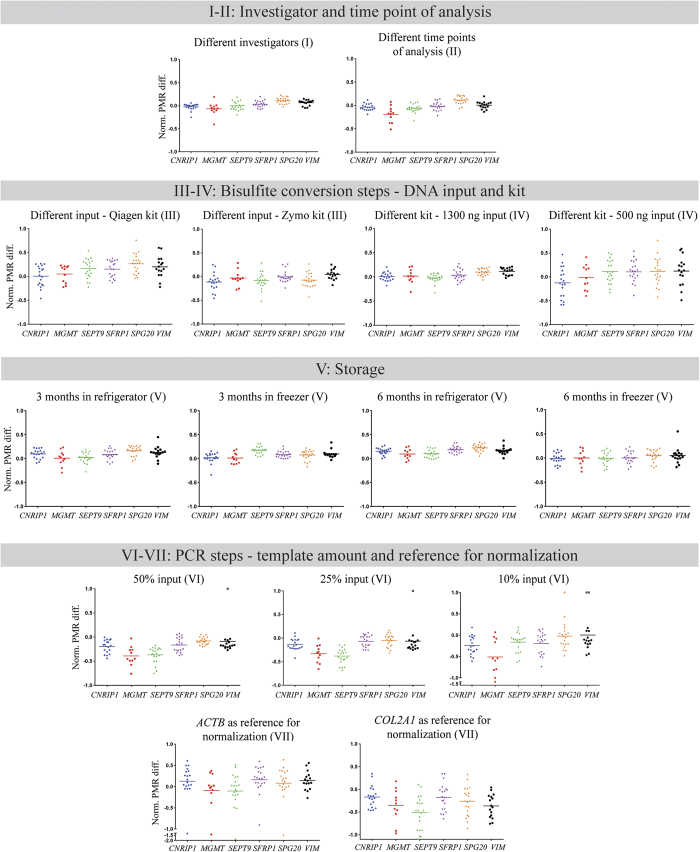
Normalized PMR differences for individual assays and cell lines, per tested parameter. Each cell line is represented by an individual dot. Each plot represents a comparison between the default and an alternative parameter. The roman numerals correspond to the pipeline in Fig. 1. Each plot for the storage experiments (V), represents an average of four rounds of analysis; two repeated rounds with both bisulfite kits (results for individual rounds are shown in Supplementary Fig. S6). Cases with a PMR_alternative_ = 0 and PMR_default _≠ 0 contributes with a normalized PMR difference of 1.0, and are indicated with an unfilled dot. Abbreviations: Norm. PMR diff., normalized PMR difference.

Over 15,000 individual PCRs were performed in the present study (see Supplementary Information) involving evaluation of various pipeline parameters across six different gene promoters and 20 colon cancer cell lines (Fig. 1). The normalized PMR differences per locus and cell line for the tested parameters are summarized in Fig. 2. The reference for normalization (VII in Figs 1 and 2) and the template amount in the PCR (VI) caused the largest normalized PMR differences. Storage (V), DNA input in the bisulfite conversion (III), and type of bisulfite kit (IV) caused intermediate levels of difference. Different investigators (I) and different time points of analysis (II) resulted in the smallest differences across the tested parameters.

**Figure 3:**
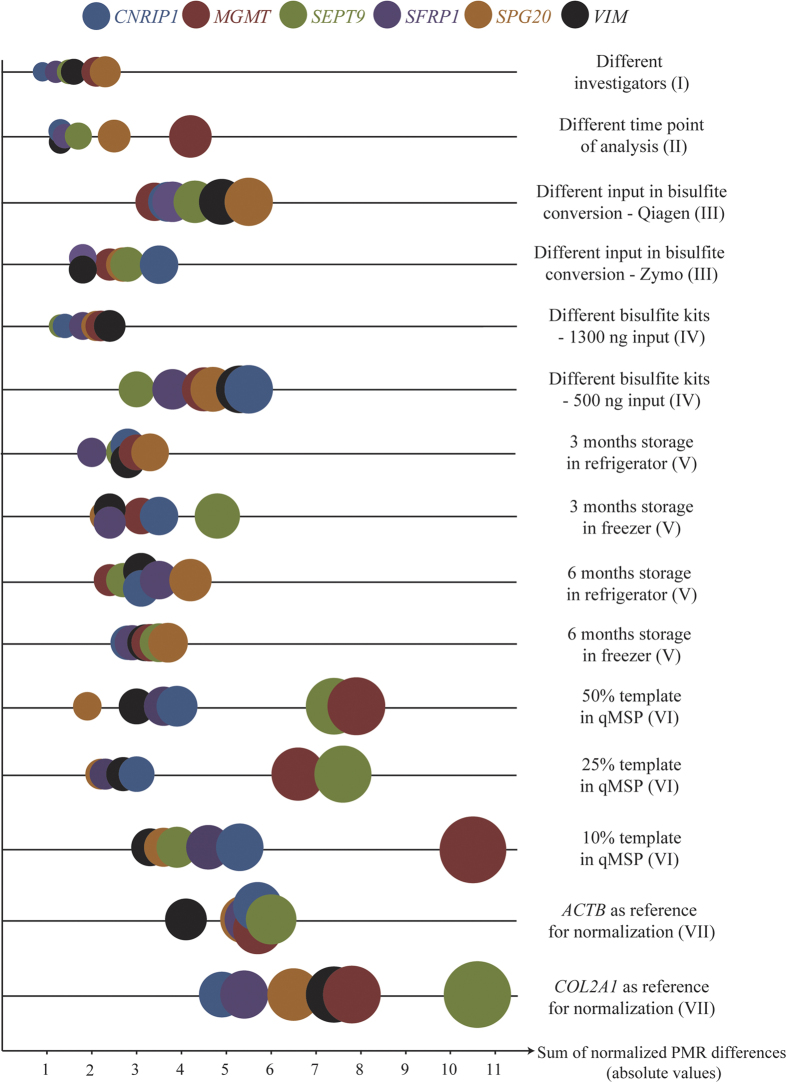
The sum of normalized PMR difference varies substantially between gene assays. The plot illustrates the sum of normalized PMR differences (using absolute values) for all cell lines per assay, and is shown for all the tested parameters. The colours symbolize the individual assays, as indicated on top. The area of each bubble represents the sum of the absolute normalized PMR difference for the respective assay, multiplied by a factor of 25 for improved visualization. The roman numerals correspond to those in Figs 1 and 2.

Interestingly, the sum of the normalized PMR differences for all cell lines (absolute values) varied substantially from gene promoter to gene promoter for several of the tested parameters (Fig. 3). *SFRP1* exhibited significantly lower normalized PMR differences across all tested parameters than expected by chance (*P *= 0.017), while *MGMT* tended to have higher normalized PMR differences (*P *= 0.048; Supplementary Table S2). Moreover, for the individual experimental parameters, the normalized PMR differences per gene assay varied with a magnitude of more than five (Supplementary Table S3). In this regard, the lowest coefficient of variation (CV) was observed for ‘6 months storage in freezer’ (CV = 0.107), and the highest for ‘25% template in qMSP’ (CV = 0.587).

**Figure 4:**
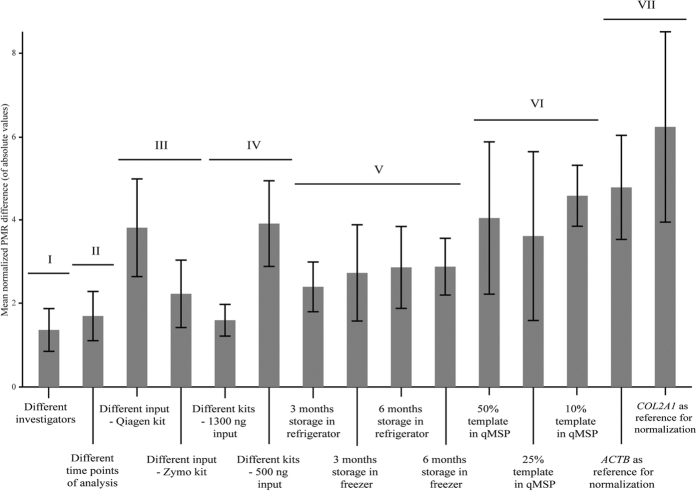
Variation in methylation level induced by alternative parameters. X-axis: tested parameters, where each column represents a comparison between the default and an alternative parameter for the same source of variability. Y-axis: the average of the sum of normalized PMR difference (absolute values) per assay. The columns are sorted according to the sources of variability and marked with roman numerals corresponding to those in Figs 1, 2 and 3. Standard deviation bars are indicated on top of each column. Each of the four columns for the storage experiments represents an average of four rounds of analysis (see legend of Fig. 2).

To identify the parameters that in total (*i.e.* across the six assays and 20 cell lines) induced the largest differences in PMR values, the average of the normalized PMR difference values (Fig. 3) was calculated. The results are shown in Fig. 4. In accordance with Fig. 2, the reference for normalization (VII) caused the highest mean normalized PMR difference, with 6.2 and 4.8 for *COL2A1* and *ACTB*, respectively. Of particular notice, use of *ACTB* caused an approximate doubling of PMR values for all assays in Colo320 cells and a halving in V9P cells, while *COL2A1* resulted in a doubling of PMR values in LS1034 cells (Supplementary Table S4). Varying the amount of template in the qMSP (VI) also caused high mean normalized PMR differences; 4.1, 3.6 and 4.6 when reducing the default template amount to 50%, 25%, and 10%, respectively. Storage of bisulfite converted DNA (V) resulted in mean normalized PMR differences between 2.4 and 2.9. Different amounts of input DNA in the bisulfite conversion reaction gave a mean normalized PMR difference of 3.8 (Qiagen kit) and 2.2 (Zymo kit). The corresponding values for different bisulfite kits (IV) were 1.6 for 1300 ng input, and 3.9 for 500 ng input. The mean normalized PMR differences for different investigators (I) and different time points of analysis (II) were the lowest among the tested parameters with 1.4 and 1.7, respectively. Overall, the magnitude of the normalized PMR difference caused by a specific parameter varied considerably (Fig. 4). Even from repeated analyses using identical parameters (represented by ‘different time points of analysis (II)’), the absolute difference between two corresponding PMR values (ΔPMR) ranged from 0 to 29, with a 95^th^ percentile of 13, a mean of five, and a median of four. In comparison, from use of variable template amount in the qMSP (*i.e.* 10% input in qMSP vs. default) the ΔPMR range was 0–62, with a 95^th^ percentile of 46, a mean of 15, and a median of 10 (Supplementary Fig. S3).

### DNA recovery after bisulfite conversion

A slightly higher recovery of bisulfite converted DNA was observed with use of the Qiagen kit compared to the Zymo kit (mean_Qia_: 18.9 ng/μl vs. mean_Zymo_: 16.5 ng/μl and median_Qia_: 16.3 ng/μl vs. median_Zymo_: 13.5 ng/μl; from a representative round of bisulfite conversion of the 20 cell lines). This corresponds to an average DNA recovery of 58% for the Qiagen kit, and 51% for the Zymo kit. Furthermore, for the Qiagen kit, the recovery of bisulfite treated DNA tended to decrease with the time from the completion of the conversion reaction, until DNA cleanup in the Qiacube. However, these variations were minor compared to the analyses with reduced template amount in the qMSP. Moreover, when a non-adjusted theoretical input of 32.5 ng bisulfite converted DNA was used as the default, the measured differences in bisulfite treated DNA concentrations did not seem to systematically affect PMR values (Supplementary Figs S4 and S5).

## Discussion

Diverging DNA methylation frequencies are commonly being reported in the literature for the same locus in the same type of disease. Based on a systematic approach and more than 15,000 individual PCRs, we demonstrate that technical variability can be introduced to various degrees at all steps of bisulfite PCR-based DNA methylation analyses. In the present study, the magnitude of normalized PMR differences varied over fourfold across the tested parameters (from 1.4 to 6.2; Fig. 4). This underscores that a highly standardized pipeline, including clearly defined experimental parameters at each step, is crucial for robust and accurate DNA methylation results.

The template amount in the PCR is critical. Clinical material is valuable and often scarce, and reducing the DNA input might be tempting. Theoretically, quantitative PCR- analyses should not be affected by varying template amounts, due to the reference for normalization. However, this is only true within a limited range. Here we show that relatively large differences in PMR values (*e.g.* from PMR 36 to PMR 73) are introduced for the same gene in the same cell line when different amounts of bisulfite treated input DNA are used (Fig. 4). Variations in template amount should therefore be avoided for samples within the same experiment.

A standardized input amount should also be used in the bisulfite conversion reaction. More consistent results were observed using higher input (1300 ng) with the Qiagen kit, and lower (500 ng) with the Zymo kit, partly corresponding to the manufacturers’ recommendations. Although 500 ng is within the recommended range for the Qiagen kit, the results were less consistent than with 1300 ng input (Fig. 4). Still, the Qiagen kit is attractive based on its ability to robustly convert larger quanta of DNA per round, the possibility of standardizing the clean-up procedure using a QIAcube, as well as the slightly higher recovery.

Storage of bisulfite converted DNA caused intermediate variation in PMR values (Fig. 4). This was seen when comparing samples analysed immediately after bisulfite conversion to samples stored in a refrigerator, and to samples stored in a freezer, the latter representing the long term storage condition as recommended from the bisulfite kit manufacturers. Interestingly, the storage-induced variation was at approximately the same level regardless of storage duration (three or six months) and temperature (freezer or refrigerator). Thus, immediate analysis is recommended; however, if samples are to be stored in a refrigerator for several months, measures should be taken to avoid evaporation, including using safe-lock tubes and parafilm sealing of these.

The choice of reference for normalization is also crucial for the resulting PMR values. In several diseases, and cancer in particular, chromosomal deletions and amplifications are frequent^15^,^16^. Consequently, use of single-/low-copy genes for normalization was disfavoured a decade ago^17^, but they are still frequently used^18^,^19^,^20^,^21^,^22^,^23^. Here we confirm that use of single-/low-copy genes such as *ACTB* and *COL2A1* can result in doubling and halving of PMR values (Supplementary Table S4) for samples with known aberrations at these loci (ref. 24 and Graue Berg, unpublished data). In contrast, the repetitive ALU element, which exists in approximately 1 million copies per haploid genome, is considerably more robust, and will as previously shown^17^ be unaffected by such aberrations.

The lowest variation in PMR values in this study was seen from analyses performed by different investigators in the same lab, as well as by the same investigator at different time points (Fig. 4). This supports the conclusion from a previous study, stating that repeated runs of bisulfite conversion followed by qMSP showed good performance and precision^25^. A certain level of variation in methylation results should however always be expected when performing bisulfite PCR-based DNA methylation analyses. This applies even when analyses are carried out in a highly standardized manner, including use of an automated pipetting station, as well as commercially purchased reagents and DNA controls, and care should be taken not to interpret such variation as biological significant.

Inclusion of six gene assays in the present study demonstrated that the magnitude of divergence in methylation results can be gene/assay-specific. This is supported by the fact that across all parameters, the analyzed genes displayed from higher to lower normalized PMR differences than expected by chance (Supplementary Table S2). Moreover, for some parameters, the six assays showed similar normalized PMR difference values, while for others, they were highly diverging (corresponding to over five-fold variation in CVs; Supplementary Table S3). Thus, assay-specific variation should be considered when interpreting DNA methylation results, and thresholds for dichotomizing samples into unmethylated and methylated categories should be conservative and robust.

In conclusion, bisulfite PCR-based DNA methylation analyses should always be performed in a highly standardized manner in order to minimize technical variability in the end results. Guidelines for more robust DNA methylation analyses include; constant input amount for all samples both in the PCR and in the bisulfite conversion reaction, use of the same bisulfite conversion kit, avoidance of storage of bisulfite converted DNA, and use of a repetitive element, *e.g.* ALU, as a reference for normalization. Finally, even with a highly standardized pipeline, a certain level of variation in methylation values must be expected and recognized when interpreting and comparing results from DNA methylation studies.

## Additional Information

**How to cite this article**: Pharo, H. D. *et al*. Experimental factors affecting the robustness of DNA methylation analysis. *Sci. Rep.* **6**, 33936; doi: 10.1038/srep33936 (2016).

## Supplementary Material

Supplementary Information
