# Supplementary material for: Experimental factors affecting the robustness of DNA methylation analysis
Source: Sci Rep. 2016 Sep 27;6:33936. doi: 10.1038/srep33936 (PMC5037394; doi:10.1038/srep33936)
Supplement: Supplementary Information [file srep33936-s1.pdf]

Experimental factors affecting the robustness of DNA methylation analysis

Heidi D. Pharo, Hilde Honne, Hege M. Vedeld, Christina Dahl, Kim Andresen, Knut Liestøl, Marine Jeanmougin, Per Guldberg, Guro E. Lind*

**corresponding author*

Supplementary Information

Supplementary Text

Calculation of the number of PCRs performed in the present study:

27 samples (including cell lines and positive controls) were analyzed in triplicates, resulting in 27 x 3 = 81 PCRs per gene assay. Each round of analysis included 7 gene assays (6 target genes, *CNRIP1*, *MGMT*, *SEPT9*, *SPG20*, *SFRP1* and *VIM* and one reference for normalization; either ALU, *ACTB* or *COL2A1*). Finally, 28 rounds of analyses were performed to test the various variables.

In total: 81 PCRs x 7 gene assays x 28 rounds of analyses = 15,876 PCRs

Supplementary Tables

Gene	Sequence accession number	Amplicon length	Sense primer	Antisense primer	Probe	Reference
ACTB	Y00474	133	TGG TGA TGG AGG AGG TTT AGT AAG T	AAC CAA TAA AAC CTA CTC CTC CCT TAA	6FAM-ACC ACC ACC CAA CAC ACA ATA ACA AAC ACA-MGB	Costa *et al.* (1)
ALU-C4	Based on consensus sequence – see reference	98	GGT TAG GTA TAG TGG TTT ATA TTT GTA ATT TTA GTA	ATT AAC TAA ACT AAT CTT AAA CTC CTA ACC TCA	6FAM-CCT ACC TTA ACC TCC C-MGB	Weisenberger *et al.* (2)
CNRIP1	NM_015463	55	TTT AGT TGC GCG GAT TTG C	GCA CCC GAA AAC TCG CTC TA	6FAM-CCG CAA ACC GCC G-MGB	Lind *et al.* (3)
COL2A1	L10347	91	TCT AAC AAT TAT AAA CTC CAA CCA CCAA	GGG AAG ATG GGA TAG AAG GGA ATAT	6FAM-CCT TCA TTC TAA CCC AAT ACC TAT CCC ACC TCT AAA-MGB	Widschwendter *et al.* (4)
MGMT	NM_002412	83	GCG TTT CGA CGT TCG TAG GT	CAC TCT TCC GAA AAC GAA ACG	6FAM-AAA CGA TAC GCA CCG CGA-MGB	Havik *et al.* (5)
SEPT9	NM_001113493	98	CGC GCG ATT CGT TGT TTA TTA	CCA ACC CAA CAC CCA CCT T	6FAM-GGA TTT CGC GGT TAA C-MGB	Ahmed *et al.* (6)
SFRP1	NM_003012	70	GAA TTC GTT CGC GAG GGA	AAA CGA ACC GCA CTC GTT ACC	6FAM-CGT CAC CGA CGC GAA-MGB	Andresen *et al.* (7)
SPG20	NM_015087	84	GCG CGT CGT GGA ACG T	CTA CGC TCG CCG AAA ACC	6FAM-CGC GCT TAC CGT AAC AA-MGB	Lind *et al.* (3)
VIM	NM_003380	106	GGT CGA GTT TTA GTC GGA GTT ACG T	CCC GAA AAC GAA ACG TAA AAA CTA	6FAM-CGT ATT TAT AGT TTG GGT AGC GC-MGB	Ahmed *et al.* (6)

Supplementary Table S1: Primer- and probe sequence information. The table includes all qMSP primer- and probe sequences used in the present study.

Reference List to Supplementary Table S1

1. Costa VL, Henrique R, Danielsen SA, Eknaes M, Patricio P, Morais A, et al. TCF21 and PCDH17 methylation: An innovative panel of biomarkers for a simultaneous detection of urological cancers. Epigenetics 2011;6:1120-30.
2. Weisenberger DJ, Campan M, Long TI, Kim M, Woods C, Fiala E, et al. Analysis of repetitive element DNA methylation by Methylight. Nucleic Acids Res 2005;33:6823-36.
3. Lind GE, Danielsen SA, Ahlquist T, Merok MA, Andresen K, Skotheim RI, et al. Identification of an epigenetic biomarker panel with high sensitivity and specificity for colorectal cancer and adenomas. MolCancer 2011;10:85.
4. Widschwendter M, Siegmund KD, Muller HM, Fiegl H, Marth C, Muller-Holzner E, et al. Association of breast cancer DNA methylation profiles with hormone receptor status and response to tamoxifen. Cancer Res 2004;64:3807-13.
5. Havik AB, Brandal P, Honne H, Dahlback HS, Scheie D, Hektoen M, et al. MGMT promoter methylation in gliomas - assessment by pyrosequencing and quantitative methylation-specific PCR. JTranslMed 2012;10:36.
6. Ahmed D, Danielsen SA, Aagesen TH, Bretthauer M, Thiis-Evensen E, Hoff G, et al. A tissue-based comparative effectiveness analysis of biomarkers for early detection of colorectal tumors. ClinTranslGastroenterol 2012;3:e27.
7. Andresen K, Boberg KM, Vedeld HM, Honne H, Hektoen M, Wadsworth CA, et al. Novel target genes and a valid biomarker panel identified for cholangiocarcinoma. Epigenetics 2012;7:1249-57.

**Gene promoter →**	***CNRIP1***	***MGMT***	***SEPT9***	***SFRP1***	***SPG20***	***VIM***
Probability of observing a higher rank sum (based on normalized PMR difference) than expected by chance	0.6673	0.0484	0.1793	0.9830	0.2747	0.8140
Probability of observing a lower rank sum (based on normalized PMR difference) than expected by chance	0.3327	0.9516	0.8207	0.0170	0.7253	0.1860

Supplementary Table S2: Evaluation of potential assay-specific variation in normalized PMR differences. The probabilities of observing a higher or lower rank sum than expected by chance are presented, and indicate that normalized PMR differences can be gene/assay-specific across the tested parameters. Of notice, a two-tailed test was performed, and the reported p-values in this table represent one side of the test (higher or lower), meaning that a value below 0.025 indicate a significant result. Consequently, *SFRP1* shows significantly lower normalized PMR difference ranks than expected by chance (*P*=0.0170), while *MGMT* show a strong tendency of having higher normalized PMR difference ranks than expected by chance (*P*=0.048).

**Investigated parameter**	**CV**
Different investigators	0.331
Different time points of analysis	0.551
Different input in bisulfite conversion - Qiagen	0.188
Different input in bisulfite conversion - Zymo	0.260
Different bisulfite kit - 1300ng	0.239
Different bisulfite kit - 500ng	0.210
3 months storage in refrigerator	0.156
3 months storage in freezer	0.313
6 months storage in refrigerator	0.199
6 months storage in freezer	0.107
50% template in qMSP	0.531
25% template in qMSP	0.587
10% template in qMSP	0.518
ACTB as reference for normalization	0.124
COL2A1 as reference for normalization	0.288

Supplementary Table S3: Evaluation of potential parameter-specific variation in normalized PMR differences. The coefficient of variation (CV; *i.e.* standard deviation of the normalized PMR differences over the mean) across the six genes/assays per parameter is shown. For some of the tested parameters, the normalized PMR differences were highly similar among the six assays (most similar for '6 months storage in freezer'; CV=0.107), while for others parameters, they were considerably more diverging (most diverging for '25% input in qMSP'; CV=0.587). Thus, more than fivefold variation in CVs was observed across the tested parameters.

**Cell lines ↓** **Genes →**	***CNRIP1***		***MGMT***		***SEPT9***		***SFRP1***		***SPG20***		***VIM***	
***ACTB* versus ALU as reference for normalization**												
	ALU	*ACTB*	ALU	*ACTB*	ALU	*ACTB*	ALU	*ACTB*	ALU	*ACTB*	ALU	*ACTB*
**Colo320** – PMR values	74	180	61	150	116	283	53	130	66	160	0	0
**V9P** – PMR values	84	40	0	0	94	45	80	38	33	16	41	19
***COL2A1* versus ALU as reference for normalization**												
	ALU	*COL2A1*	ALU	*COL2A1*	ALU	*COL2A1*	ALU	*COL2A1*	ALU	*COL2A1*	ALU	*COL2A1*
**LS1034** – PMR values	47	90	36	68	71	136	37	72	37	70	61	116

Supplementary Table S4: The effect of chromosomal aberrations on PMR values with use of single-copy genes as normalization references. The table gives an overview of cell lines with a doubling (Colo320 and LS1034) or a halving (V9P) of PMR values when *ACTB* or *COL2A1* were used as reference genes.

Supplementary Figures

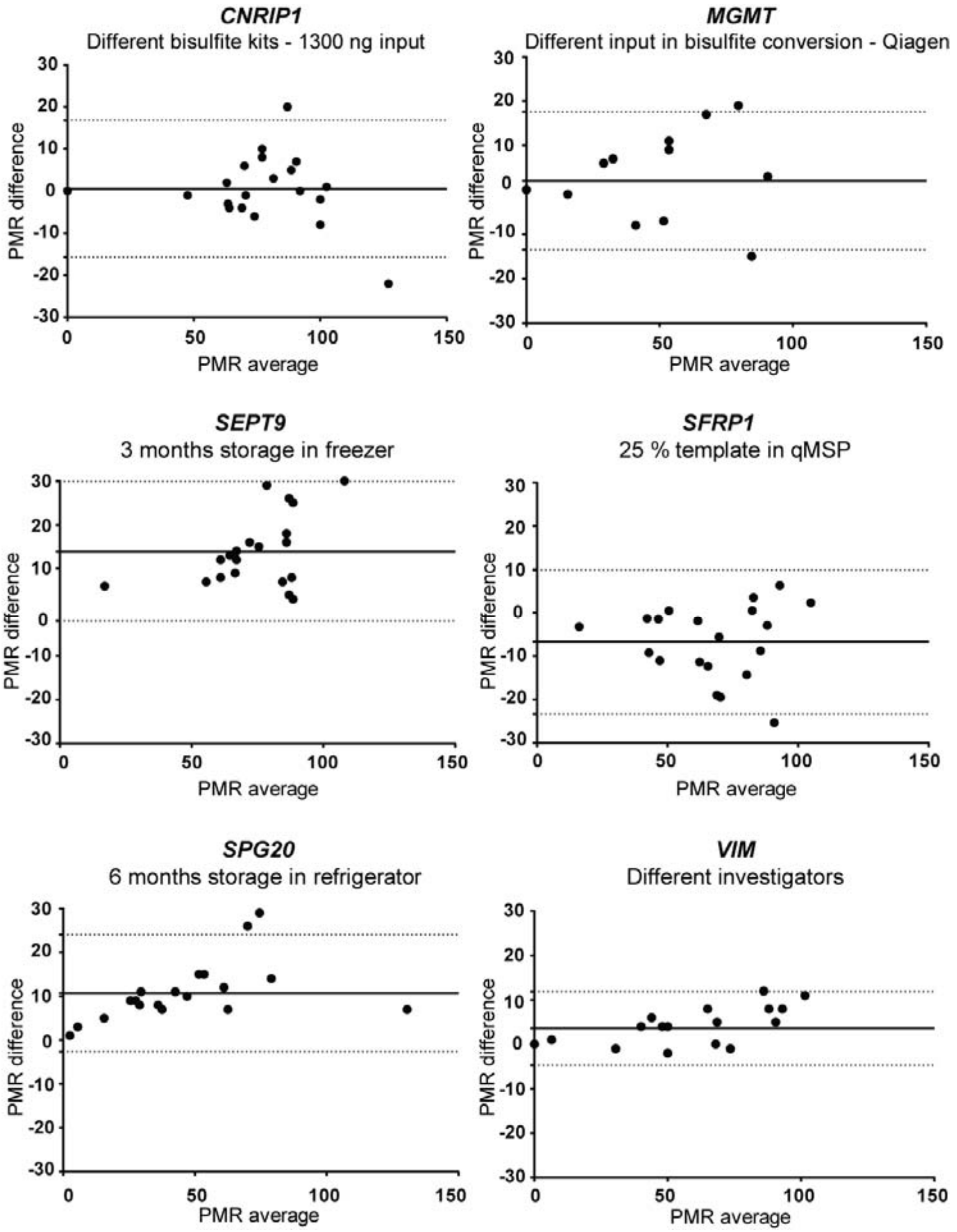

Supplementary Figure S1: Bland-Altman plots showing PMR difference against PMR average per cell line for the six gene promoters included in the study. X-axis: the average of two PMR values for the same cell line; from testing a default and an alternative parameter. Y–axis: the difference between the two PMR values. Each plot represents one gene assay, and is a representative example from a comparison of a default and an alternative parameter in the pipeline (Fig. 1 in the main text). Each dot represents one cell line. The solid line indicates the average of the differences, and the dotted lines the average ±1.96 times its standard deviation. For two of the plots, outliers are present, but omitted from the figure as they are outside the axis limits (more specific information can be provided upon request). As evident from the plots, the PMR differences are increasing with higher PMR average for all gene assays, indicating that that higher PMR values typically cause higher PMR differences.

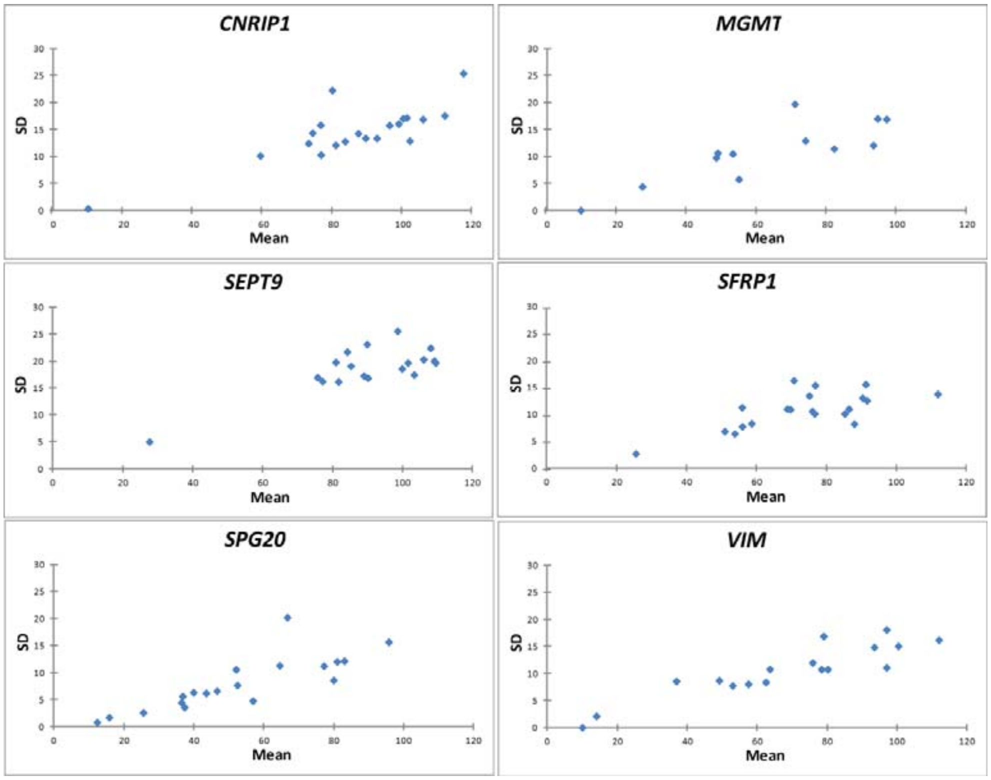

Supplementary Figure S2: 'Standard deviation versus Mean'-plots for the six gene promoters included in the study. X-axis: Mean of PMR values. Y-axis: Standard deviation (SD) of PMR values (from all tested parameters). Each plot represents one gene assay, and each point represents one cell line. For two of the plots, outliers are present, but omitted from the figure as they are outside the axis limits (more specific information can be provided upon request). A linear correlation tendency is seen for all genes, confirming the finding from the Bland-Altman plots (Supplementary Figure S1), that higher PMR values typically cause higher PMR differences.

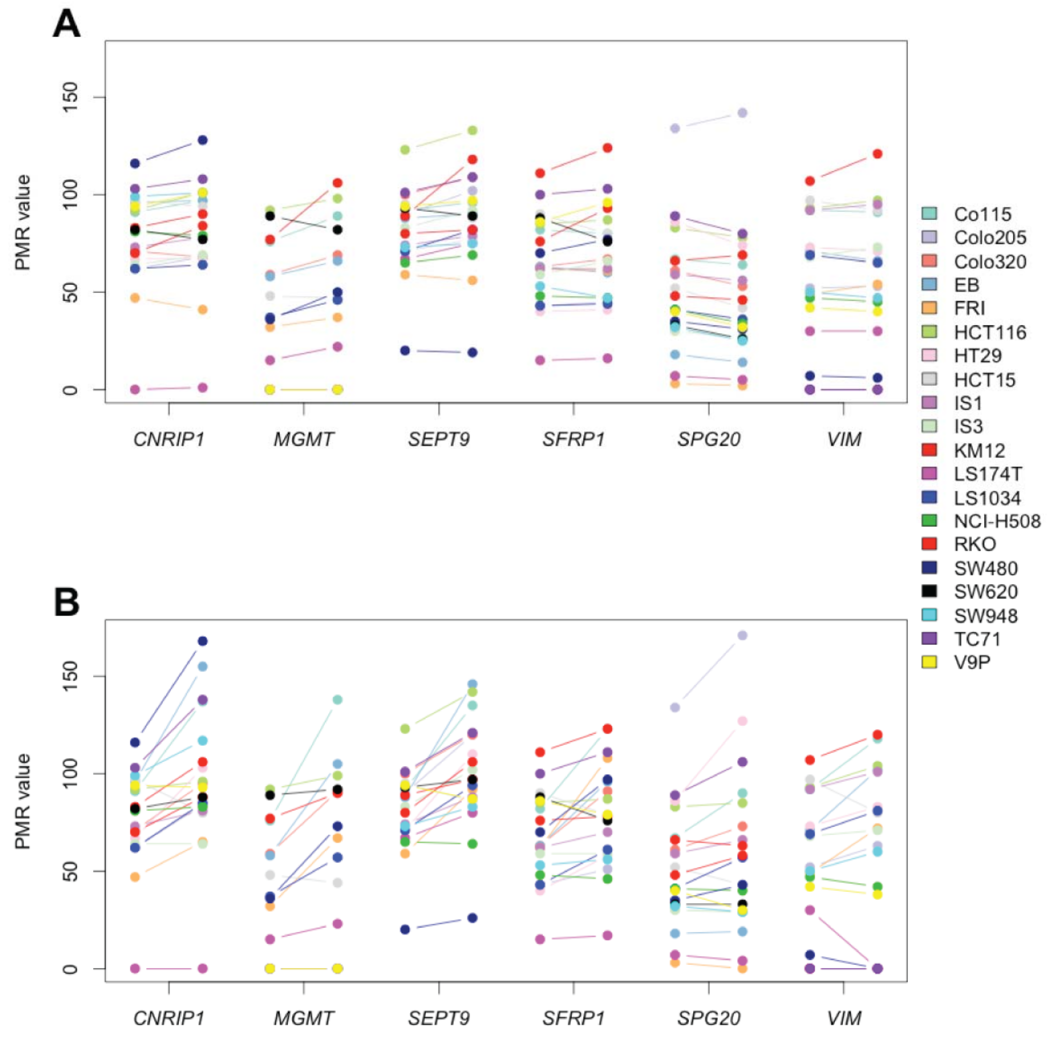

Supplementary Figure S3: Differences in PMR values resulting from standardized and non-standardized analyses. X-axis: gene assays. Y-axis: PMR values. Two connected dots represent PMR values for the same cell line when comparing results from two rounds of standardized analyses using identical parameters (A) and from analyses using varying parameters, here illustrated by '10% input in qMSP (VI)' (B).

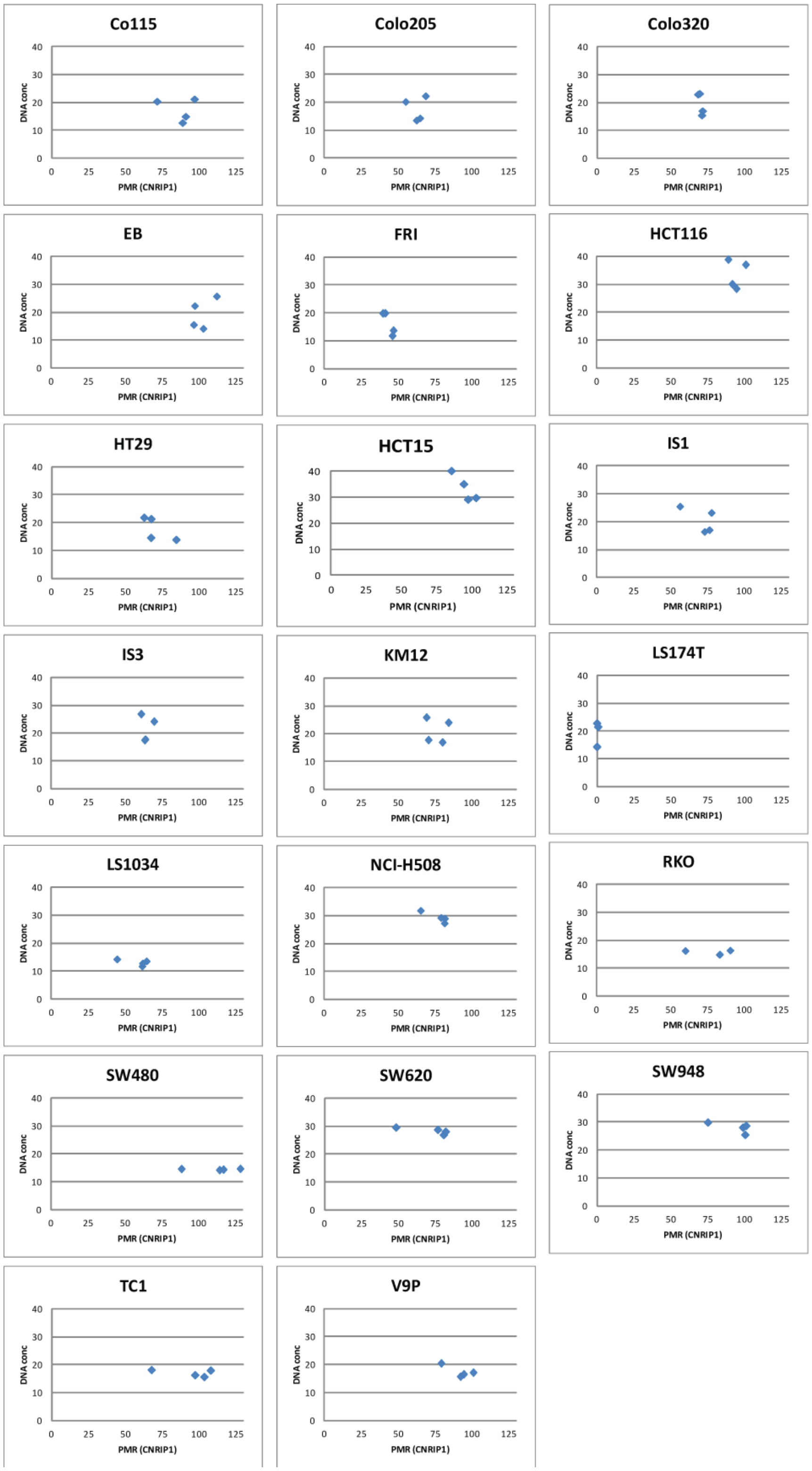

Supplementary Figure S4: Correlation plots showing DNA concentration after bisulfite conversion against corresponding PMR value for a representative assay (*CNRIP1*). X-axis: PMR value for *CNRIP1* in a given cell line. Y-axis: DNA concentration after bisulfite conversion (measured on the NanoDrop1000 instrument) for the same cell line. Each of the plots represents one cell line (as indicated in the plot title). The four dots in each plot represent four rounds of analysis where the same parameters have been used at each step of the pipeline (the default parameters), thus basically 'identical' rounds of analysis. No clear correlation between DNA concentration and PMR value can be seen.

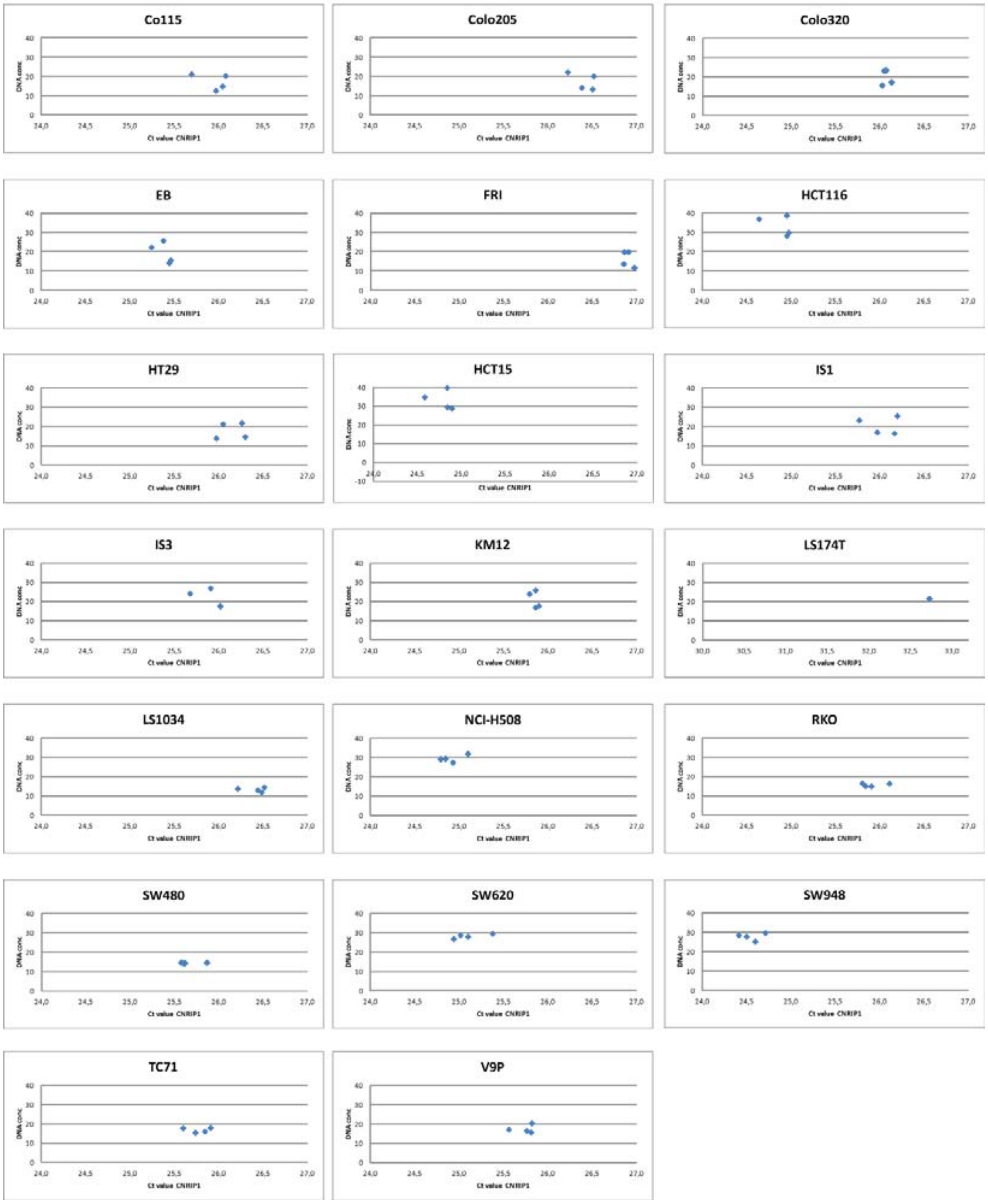

Supplementary Figure S5: Correlation plots showing DNA concentration after bisulfite conversion against corresponding Cq value for a representative assay (*CNRIP1*). X-axis: C_q value for *CNRIP1* in a given cell line. Y-axis: DNA concentration after bisulfite conversion (measured on the Nanodrop1000 instrument) for the same cell line. Each of the plots represents one cell line (as indicated in the plot title). The four dots in each plot represent four rounds of analysis where the same parameters have been used at each step of the pipeline (the default parameters), thus basically ‘identical’ rounds of analysis. No clear correlation between DNA concentration and C_q value can be seen.

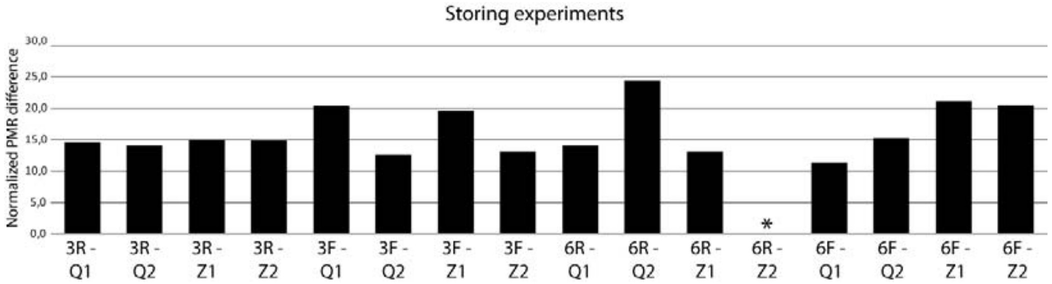

Supplementary Figure S6: Normalized PMR differences for individual rounds of analysis related to storing as source of variability. The normalized PMR differences for all cell lines and all asssays (absoulte values) per tested storing parameter have been summed. ”Three months storage in a refrigerator” results in quite consistent results. The other storing conditions showed a somewhat more variable level of normalized PMR differences across first and second round of analysis, as well as across the two different bisulfite kits. *This round of analysis has been censored due to evaporation during storage and thus insufficient amount of template left for downstream qMSP. Abbreviations: 1 = 1. round of analysis; 2 = 2. round of analysis; 3 = 3 months storage; 6 = 6 months storage; F = freezer; R = refrigerator; Q = Qiagen kit, Z = Zymo.
